# Prevalence of Stroke‐Related Sarcopenia: A Systematic Review and Meta‐Analysis

**DOI:** 10.1002/pri.70134

**Published:** 2025-12-11

**Authors:** Lavinia Pereira dos Santos, Bruna Machado Staico, Anna Balsamão Vaz, Ana Carolina Joviano Galvão, Ana Luiza Miranda de Oliveira, Maria Clara Ferreira, Amanda Leopoldino, Janaíne Cunha Polese

**Affiliations:** ^1^ Faculdade de Ciências Mêdicas de Minas Gerais Belo Horizonte Brazil; ^2^ Universidade Federal de Minas Gerais Belo Horizonte Brazil

**Keywords:** prevalence, sarcopenia, stroke, systematic review

## Abstract

**Background and Purpose:**

Sarcopenia can lead to negative outcomes for the functionality of individuals post‐stroke. Therefore, understanding the prevalence of sarcopenia after a stroke is extremely important in order to plan specific interventions. To provide an up‐to‐date meta‐analysis to investigate the prevalence of sarcopenia in individuals after stroke.

**Methods:**

This systematic review was carried out in the MEDLINE, SCIELO, EMBASE, LILACS, PEDro, EBSCOHOST, AMED and gray literature databases, with no date or language restrictions.

**Results:**

Of the 1142 articles found, 31 were included in the review, totaling 7357 post‐stroke individuals. Of these, 74.1% were in an in‐hospital setting and 38.7% were in the acute post‐injury phase. Most of the studies were carried out in Asia (80.6%) and diagnosed sarcopenia according to the Asian Working Group of Sarcopenia consensus (58%). A low‐quality screening prevalence of 42.4% (95% CI 13.0%–78.5%; *I* ^2^ = 99%) and 37.2% (95% CI 30.7%–44.2%; *I* ^2^ = 97%) of sarcopenia diagnoses with moderate‐quality evidence were observed.

**Discussion:**

High prevalence rates of post‐stroke sarcopenia were observed, with high variability in relation to the form used for diagnosis and screening. It is important to highlight additional points observed such as a lack of consensus on definition and diagnostic criteria for sarcopenia, a variation of tools to measure body mass, a lack of consensus regarding the side of the assessment and a high heterogeneity of the samples analyzed, which can directly impact on the results found.

## 1 Introduction

In recent years, stroke has shown a global prevalence of 89.13 million cases, considering all stroke subtypes (Tsao et al. 2023). The disease can trigger various systemic consequences that last and worsen over the years after stroke, such as reduced functioning, cognitive capacity, and quality of life (Tsao et al. 2023). Individuals after stroke frequently present muscle weakness, which is associated with various muscle abnormalities, such as denervation, disuse, inflammation, remodeling, and spasticity (Scherbakov et al. 2013). Stroke increases the release of pro‐inflammatory cytokines and cellular oxidative stress, which promotes proteolysis and muscle degradation (Carda et al. 2013; Cruz‐Jentoft et al. 2019). Additionally, muscle mass can also be reduced due to the disuse and immobilization of the paretic limb, which are very common in individuals after stroke (Carda et al. 2013). Post‐stroke patients presented a 20%–24% loss of cross‐sectional area and volume on the paretic side compared to the non‐paretic side (Ryan et al. 2017). Sarcopenia is the age‐related reduction in lean mass, which can result in a reduction in strength, metabolic rate and aerobic capacity, and therefore functional capacity (Evans 2010). When related to a disease such as stroke, it is considered as a secondary sarcopenia and can trigger muscle atrophy more quickly, thus becoming part of the disease process (Li et al. 2020). However, this association has not yet been widely studied (Kortebein et al. 2007; Botö et al. 2021).

The screening for sarcopenia is fundamental, so individuals can be adequately evaluated and receive early treatments, aiming for the best possible outcomes (Bahat et al. 2022). For this reason, the main guidelines are defined by the European Working Group on Sarcopenia in Older People (EWGSOP), the International Working Group on Sarcopenia (IWGS), the Asian Working Group for Sarcopenia (AWGS), and the American Foundation for the National Institutes of Health (FNIH), which suggest the cut‐off point for aspects related to screening for sarcopenia, such as muscle weakness (Gao et al. 2015; Patel et al. 2013; Lera et al. 2017; Yang et al. 2018). According to the latest European consensus on the diagnosis of sarcopenia (EWGSOP 2), it can be identified using simple parameters such as handgrip strength to identify general muscle strength, body mass index to detect muscle mass, and physical performance tests such as the Timed‐Up and Go Test (TUG) to measure function, thus comprising the three variables that encompass sarcopenia (Cruz‐Jentoft et al. 2019).

A previous systematic review investigated the prevalence of sarcopenia in individuals after stroke and included studies with considerably different screening standards, which further illustrates this unequal assessment, consequently impacting the clinical practice of individuals after stroke (Su et al. 2020). It was found a 42% (95% confidence interval: 33%–52%, *I* ^2^ = 91%) polled prevalence of sarcopenia. In this previous systematic review, relevant data such as demographic aspects, stroke characteristics and definition of sarcopenia were taken into consideration (Su et al. 2020). However, this study presented some gaps that should be considered, such as measures like handgrip strength on the paretic or non‐paretic side and the prevalence of sarcopenia screening, which are relevant factors for clinical practice and research, were not addressed. Furthermore, on the previous systematic review (Su et al. 2020), only studies in English were included. Therefore, the aim of this study was to provide an up‐to‐date meta‐analysis to investigate the prevalence of sarcopenia in individuals after stroke.

## 2 Methods

The protocol of this systematic review was registered at PROSPERO (CRD42023416980). Joanna Briggs Institute Reviewers' Manual (The Systematic Review of Prevalence and Incidence Data) (Munn et al. 2014), the Cochrane Collaboration (Higgins and Green 2023) and MOOSE (Meta‐analysis of Observational Studies in Epidemiology) (Stroup et al. 2000) reporting guidelines were followed.

## 3 Data Sources and Search Strategy

Systematic searches were conducted in electronic databases, including MEDLINE, SCIELO, EMBASE, LILACS, PEDro, EBSCOHOST, AMED and gray literature without date (from inception until May 2024) or language restrictions. The search strategy included the following keywords: “epidemiology” “prevalence” “stroke” “sarcopenia” “muscle loss” “low muscle mass” “appendicular lean mass” “muscle weakness” and “muscle atrophy” (Su et al. 2020). In addition, the Boolean terms “OR” (within columns) and “AND” (between columns) were combined.

### 3.1 Inclusion and Exclusion Criteria

Eligibility criteria included RCTs, cohort studies, case‐control, and cross‐sectional studies that investigated individuals after stroke aged ≥ 18 with data available for sarcopenia, or any other combinations of physical capability markers called sarcopenia. No restriction regarding the date of publication or language was used. Studies with associated comorbidities other than stroke and with missing data on how sarcopenia was diagnosed were excluded.

### 3.2 Screening of the Literature Search Results

The article titles and abstracts were reviewed by two independent researchers for eligibility according to the inclusion and exclusion criteria using the software Rayyan (Dos Reis et al. 2023). If the study was deemed suitable, it was moved onto the next phase, involving retrieval of the full text. After the review of the full text, if the article was still considered suitable, then it was included for data extraction. Although a protocol was created to avoid handling disagreements, such as data collection form, a third reviewer solved any disagreements. Duplicate publications were removed to decrease the risk of bias. Additionally, for any missing information, the corresponding author was contacted by email. Studies were excluded if the corresponding authors would not answer back after 15 days.

### 3.3 Data Extraction and Outcome Measures

The following information was collected as part of the data extraction procedure.

– First author and date of publication;
– Study design, setting and sample size (related to stroke individuals);
– Socio‐demographics characteristics: age, sex, country and setting where the study was conducted;
– Anthropometrics characteristics: heights, weight and body mass index;
– Stage after stroke (acute, subacute or chronic);
– Definition of sarcopenia and severe sarcopenia, and the criteria used to define it.

Two reviewers independently assessed the risk of bias for each included study using a prevalence study critical appraisal tool (The Joanna Briggs Institute approach for systematic reviews) that includes nine items that provide an assessment of the methodological quality of prevalence studies. A third reviewer solved potential disagreements (Munn et al. 2015).

### 3.4 Statistical Analysis

Descriptive statistics were used to first describe the extracted data. The prevalence of sarcopenia was described as percentages with confidence intervals (CIs). Meta‐analyses were conducted when sufficient homogeneity between studies were found.

Prevalence rates were managed as a logit event estimate to normalize the distribution of data. Logit event estimates were then back‐transformed into proportions with 95% CI when pooled analysis was carried out. The overall prevalence rates were reported in percentages. *I* ^2^ was used and low heterogeneity was considered if *I* ^2^ is < 50% and moderate to high if *I* ^2^ ≥ 50%. For *I* ^2^ < 50%, fixed effect model was used during the meta‐analysis, while for *I* ^2^ ≥ 50% random effects model was used (Higgins and Thompson 2002).

Data was analyzed using descriptive statistics and using Comprehensive Meta‐Analysis version 2.2.04 (Biostat, Englewood, NJ, USA) and SPSS version 19.0 (SPSS, Chicago, IL, USA).

The GRADE (Grading of Recommendations Assessment, Development and Evaluation) recommendations were used to grade the quality of evidence and the strength of health recommendations (Terracciano et al. 2010). Thus, for each outcome it was downgraded by one point when the following pre‐specified criteria were not met: risk of bias (< 5 out of 9), inconsistency of estimates among or within studies, imprecision for small sample sizes (< 600 participants pooled), and presence of publication bias. The GRADE system rating for the evidence set ranged from high to very low quality. Indirectness was not downgraded in the present review, since use of validated screening or diagnostic tools was a criterion of inclusion in the present study (Diz et al. 2017).

## 4 Results

### 4.1 Literature Search Results and Study Selection

**FIGURE 1:**
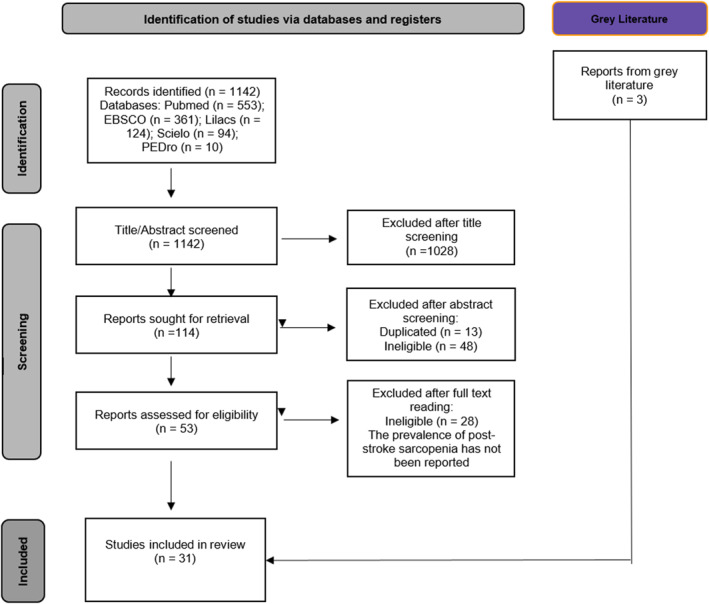
PRISMA flow diagram showing the process of study selection.

Initially, a total of 1142 articles were identified in the databases and uploaded on Rayyan software. Subsequently, at the title screening stage, 1028 were excluded due to disagreement with the third reviewer, leaving 114 articles for abstract screening. After 61 references were excluded, 13 of which were duplicates and 48 ineligible studies, 53 studies were evaluated by full text between five independent reviewers who read the studies and then exchanged them to make corrections and reach a consensus. In addition, three articles were included from the gray literature. Finally, 31 studies were included in this review (Figure 1). All the authors of the articles that contained missing data and were potentially relevant to the analysis were contacted by email.

### 4.2 Methodological Quality

The average methodological quality of the included studies was 7.8 out of 9 (range 6–9). Item 3 of the methodological quality assessment was not completed for most included studies (77% of studies). The items 1, 6, and 8 was followed by 100% of the included studies (Table 1). These items refer to subgroup analyzes (e.g., gender and age group).

**TABLE 1:** Methodological quality of the 31 included studies.

Study	1	2	3	4	5	6	7	8	9	Overall score 0–9
Abe et al. (2023)	Y	Y	N	Y	Y	Y	Y	Y	Y	8
Abe et al. (2024)	Y	Y	N	Y	Y	Y	Y	Y	Y	8
Bellelli et al. (2018)	Y	Y	N	Y	Y	Y	Y	Y	Y	8
Chang et al. (2020)	Y	Y	N	Y	Y	Y	U	Y	U	6
Galvão et al. (2024)	Y	Y	Y	N	Y	Y	Y	Y	Y	8
Imamura et al. (2023)	Y	Y	N	Y	Y	Y	Y	Y	Y	8
Inoue et al. (2021)	Y	Y	N	Y	Y	Y	Y	Y	Y	8
Jang et al. (2020)	Y	Y	N	Y	Y	Y	Y	Y	Y	8
Landi et al. (2012)	Y	Y	N	Y	Y	Y	Y	Y	Y	8
Li et al. (2022)	Y	U	N	U	Y	Y	Y	Y	Y	7
Liu et al. (2020)	Y	Y	N	Y	Y	Y	Y	Y	Y	8
Maeda and Akagi (2017)	Y	Y	N	Y	Y	Y	Y	Y	Y	8
Matsushita et al. (2019)	Y	Y	N	Y	Y	Y	U	Y	Y	7
Matsushita et al. (2020)	Y	N	Y	Y	Y	Y	Y	Y	U	7
Mohammed and Li (2022)	Y	Y	N	Y	Y	Y	U	Y	Y	7
Nozoe et al. (2024)	Y	Y	Y	N	Y	Y	Y	Y	Y	8
Ogino et al. (2024)	Y	Y	Y	Y	Y	Y	Y	Y	Y	9
Park et al. (2014)	Y	Y	N	Y	U	Y	Y	Y	Y	7
Park et al. (2019)	Y	Y	N	Y	Y	Y	Y	Y	Y	8
Ryan et al. (2017)	Y	Y	N	Y	Y	Y	Y	Y	Y	8
Sato et al. (2022)	Y	Y	Y	Y	Y	Y	Y	Y	Y	9
Shimizu et al. (2022)	Y	Y	N	Y	Y	Y	Y	Y	Y	8
Shiraishi et al. (2018)	Y	Y	N	Y	Y	Y	Y	Y	Y	8
Vahlberg et al. (2016)	Y	Y	N	Y	Y	Y	Y	Y	Y	8
Wong et al. (2022)	Y	Y	N	Y	Y	Y	Y	Y	Y	8
Yao et al. (2022)	Y	Y	Y	Y	Y	Y	Y	Y	Y	9
Yoshimura, Bise, et al. (2018)	Y	Y	Y	Y	Y	Y	Y	Y	Y	9
Yoshimura, Wakabayashi, et al. (2018)	Y	Y	N	Y	Y	Y	Y	Y	Y	8
Yoshimura et al. (2022a)	Y	Y	N	Y	Y	Y	U	Y	Y	7
Yoshimura et al. (2022b)	Y	Y	N	Y	Y	Y	Y	Y	Y	8
Yu et al. (2014)	Y	Y	N	Y	Y	Y	Y	Y	Y	8

*Note:* (1) Was the sample frame appropriate to address the target population? (2) Were study participants sampled in an appropriate way? (3) Was the sample size adequate? (4) Were the study subjects and the setting described in detail? (5) Was the data analysis conducted with sufficient coverage of the identified sample? (6) Were valid methods used for the identification of the condition? (7) Was the condition measured in a standard, reliable way for all participants? (8) Was there appropriate statistical analysis? (9) Was the response rate adequate, and if not, was the low response rate managed appropriately?

Abbreviations: N = no; U = unclear; Y = yes.

### 4.3 Study Characteristics

The investigation of the prevalence of sarcopenia after stroke included a total of 7357 individuals. Of these studies, most individuals were in an in‐hospital environment, including wards (74.1%); 12.9% in an outpatient environment; and 12.9% of the sample was in the general community. In addition, the post‐stroke phase in which the individuals were found was also analyzed: 38.7% in the acute and subacute phase; 22.5% chronic; 6.4% with individuals in the 3 phases (acute or subacute or chronic); and 32.2% did not inform the post‐stroke phase in which the prevalence of sarcopenia was surveyed. The characteristics of the included studies are summarized in Table 2.

**TABLE 2:** Characteristics of the 31 included studies.

Study	Design	Height/weight/body mass index	Stage after stroke (acute, subacute or chronic)	Setting	Participants	Criteria for sarcopenia screening or diagnosis	Criteria for severe sarcopenia	Side of assessment	Prevalence
Abe et al. (2023)	Retrospective cohort	BMI (stroke population): 23.3 ± 3.9	Acute	Hospital	*n* = 308	AWGS	—	Non‐paretic	32.4%
Abe et al. (2024)	Retrospective cohort	—	Acute	Hospital	*n* = 228	AWGS		Non‐paretic	24.6%
Bellelli et al. (2018)	Cross‐sectional	BMI (general population): < 18.5: 15 individuals (sarcopenic), 7 individuals (non sarcopenic)	—	Hospital	*n* = 70	EWGSOP	—	Non‐paretic	44.2%
		Between 18.5 e 25: 106 individuals (sarcopenic), 130 individuals (non sarcopenic)							
		> 25: 78 individuals (sarcopenic), 225 individuals (non sarcopenic)							
Galvao et al. (2024)	Cross‐sectional	—	Chronic	Community	*n* = 54	SARC‐F	—	—	21.0%
Imamura et al. (2023)	Prospective cohort	BMI (general population): 22.6	Acute	Hospital	*n* = 290	SARC‐F	—	Not informed	80.0%
Inoue et al. (2021)	Retrospective transversal	BMI (stroke population): 21.3 ± 19.6–23.7	—	Hospital	*n* = 256	AWGS	—	Non‐paretic	63.7%
Jang et al. (2020)	Retrospective cohort	BMI (stroke population): 24.2 ± 3.4	Chronic	Hospital	*n* = 194	AWGS	—	Both	41.8%
Landi et al. (2012)	Prospective cohort	BMI (general population):	—	Community	*n* = 4	EWGSOP	—	Non‐paretic	50.0%
		Sarcopenic: 23.8 ± 4.2							
		Non sarcopenic: 26.9 ± 4.3							
Li et al. (2022) (1)	Cross sectional	•	Chronic	Hospital	*n* = 28	AWGS	Low mass and low strength and low physical performance	Paretic	86.0%
Li et al. (2022) (2)	Cross sectional	—	Chronic	Hospital	*n* = 28	AWGS	Low mass and low strength and low physical performance	Non‐paretic	25.0%
Li et al. (2022) (3)	Cross sectional	—	Chronic	Hospital	*n* = 28	AWGS	Low mass and low strength and low physical performance	Not informed	75.0%
Li et al. (2022) (4)	Cross sectional	—	Chronic	Hospital	*n* = 28	EWGSOP	Low strength and low mass and low physical performance (gait speed ≤ 0.8 m/s or TUG ≥ 20 s)	Paretic	89.0%
Li et al. (2022) (5)	Cross sectional	—	Chronic	Hospital	*n* = 28	EWGSOP	Low strength and low mass and low physical performance (gait speed ≤ 0.8 m/s or TUG ≥ 20 s)	Non‐paretic	18.0%
Liu et al. (2020)	Not reported	BMI (stroke population):	—	Hospital	*n* = 45	AWGS	—	Non‐paretic	33.3%
		Non sarcopenia: 27.40 ± 2.04							
		Pre sarcopenia: 25.25 ± 2.30							
		Sarcopenia: 23.57 ± 2.51							
Maeda and Akagi (2017)	Cross‐sectional	BMI (general population): 20.65 ± 3.68	—	Hospital	*n* = 194	AWGS	—	Non‐paretic	67.0%
Matsushita et al. (2019)	Retrospective	BMI (stroke population): 22.4 ± 3.4	Acute	Hospital	*n* = 267	EWGSOP and AWGS	—	Non‐paretic	48.3%
Matsushita et al. (2020)	Cross‐sectional	BMI (stroke population):	Acute to chronic	Outpatient	*n* = 367	AWGS	—	Non‐paretic	60.6%
		Normal: 21.1 ± 2.0							
		Obese: 24.9 ± 2.8							
		Sarcopenic: 19.1 ± 2.3							
Mohammed and Li (2022)	Cross‐sectional	(Stroke population)	Chronic	Outpatient	*n* = 395	AWGS/SARC‐F		Non‐paretic	15.6%/65.8%
		Group 1 (Egyptian population): BMI: 25.3 ± 4.20							
		Weight: 71.62 ± 11.0							
		Height: 167.60 ± 8.1							
		Group 2 (Chinese population): BMI: 24.0 ± 3.04							
		Weight: 69.9 ± 12.12							
		Height: 170.0 ± 7.26							
Park et al. (2014)	Cross‐sectional	•	—	Hospital	*n* = 273	Janssen	—	Not informed	49.4%
Park et al. (2019)	Cross‐sectional	•	Subacute	Community	*n* = 39	AWGS	Appendicular skeletal muscle mass/body weight < 1 (moderate) or 2 (severe)	Non‐paretic	30.8%
Ryan et al. (2017) (1)	Cohort study	BMI (stroke population): 28.6 ± 0.4	Chronic	Outpatient	*n* = 190	ALM/h^2^	—	Not informed	16.8%
		Weight (stroke population): 82.2 ± 1.2							
Ryan et al. (2017) (2)	Cohort study	BMI (stroke population): 28.6 ± 0.4	—	Outpatient	*n* = 190	EWGSOP	—	Not informed	14.3%
		Weight (stroke population): 82.2 ± 1.2							
Ryan et al. (2017) (3)	Cohort study	BMI (stroke population): 28.6 ± 0.4	—	Outpatient	*n* = 190	IWG	—	Not informed	16.7%
		Weight (stroke population): 82.2 ± 1.2							
Ryan et al. (2017) (4)	Cohort study	BMI (stroke population): 28.6 ± 0.4	—	Outpatient	*n* = 190	ALM	—	Not informed	17.9%
		Weight (stroke population): 82.2 ± 1.2							
Sato et al. (2022)	Retrospective observational	BMI (stroke population): 23.2 (20.5–25.5)	Acute	Hospital	*n* = 211	AWGS	—	Non‐paretic	30.8%
Shimizu et al. (2022)	Cross‐sectional	BMI (stroke population):	Acute	Hospital	*n* = 443	EWGSOP	—	Not informed	62.1%
		Dysphagia level 3: 20.8 ± 0.3							
		Dysphagia level 4: 19.8 ± 3.4							
		Dysphagia level 5: 21.5 ± 2.9							
		Dysphagia level 6: 22.1 ± 3.5							
		Dysphagia level 7: 23.4 ± 3.8							
Shiraishi et al. (2018)	Cross‐sectional	BMI (stroke population): 21.9 ± 3.7	Acute	Hospital	*n* = 202	AWGS	—	Non‐paretic	53.5%
Vahlberg et al. (2016)	Cross‐sectional cohort	BMI (stroke population): 27.3 ± 4.1	Chronic	Hospital	*n* = 134	EWGSOP	—	Not informed	7.0%
Wong et al. (2022)	Cross‐sectional	BMI (stroke population): 24.53 ± 4.71 (60–69 years old)	Subacute and chronic	Hospital	*n* = 196	AWGS	—	Non‐paretic	42.3%
		23.08 ± 4.48 (> 70 years old)							
Yao et al. (2022)	Case‐control observational	BMI (stroke population): 22.97 ± 3.9	Acute	Hospital	*n* = 259	AWGS	—	Non‐paretic	46.7%
Yoshimura, Bise, et al. (2018)	Cross‐sectional	BMI (stroke population): 21.9 ± 3.8	—	Hospital	*n* = 233	AWGS	—	Not informed	53.6%
Yoshimura, Wakabayashi, et al. (2018)	Retrospective cohort	BMI (stroke population): 22.1 ± 2.5	—	Hospital	*n* = 204	AWGS	—	Non‐paretic	39.7%
Yoshimura et al. (2022a)	Retrospective cohort	BMI (stroke population): 21.2 (18.7–22.7)	Acute	Hospital	*n* = 598	AWGS	—	Non‐paretic	50.5%
Yoshimura et al. (2022b)	Retrospective cohort	BMI: 22.5 (20.2, 25.1)	Acute	Hospital	*n* = 760	ESPEN and EASO/BMI	—	Non‐paretic	4.5%/13.9%
Yu et al. (2014)	Longitudinal observational	BMI: 23.7	—	Community	*n* = 175	EWGSOP	—	Both	8.0%
Nozoe et al. (2024)	Single‐center prospective observational	BMI (stroke population): 23.8 (21.4–26.3)	—	Hospital	*n* = 247	AWGS	—	Non‐ paretic	28.0%
Ogino et al. (2024)	Cohort	BMI (stroke population): 22.3 (20.0–24.7)	Acute	Center	*n* = 456	AWGS	—	—	58.0%

Abbreviations: ALM = appendicular lean mass; AWGS = Asian Working Group for Sarcopenia; BMI = body mass index; EASO = European Association for the Study of Obesity; ESPEN = European Society for Clinical Nutrition and Metabolism; EWGSOP = European Working Group on Sarcopenia in Older People; IWG = International Working Group on Sarcopenia.

Most of the studies were carried out on the Asian continent, 80.6% (25 articles), and the rest were carried out in America, 9.6% (three articles), in Europe, 6.4% (two articles), and one study that included individuals from Asia and Africa, 3.2%; indicating a disparity in research on sarcopenia around the world.

### 4.4 Prevalence of Sarcopenia

**FIGURE 2:**
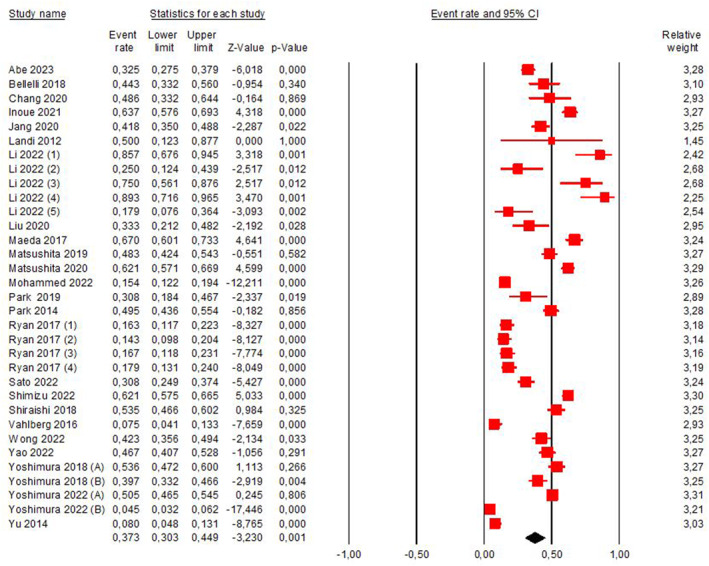
The pooled prevalence of stroke‐related sarcopenia, considering the diagnosis. Li et al. (2022) (1): criteria used for sarcopenia diagnosis: AWGS; Assessed side: paretic. Li et al. (2022) (2): criteria used for sarcopenia diagnosis: AWGS; Assessed side: non‐paretic. Li et al. (2022) (3): criteria used for sarcopenia diagnosis: AWGS; Assessed side: not informed. Li et al. (2022) (4): criteria used for sarcopenia diagnosis: EWGSOP; Assessed side: paretic. Li et al. (2022) (5): criteria used for sarcopenia diagnosis: EWGSOP; Assessed side: non‐paretic. Ryan et al. (2017) (1): criteria used for sarcopenia diagnosis: Appendicular lean mass/height^2^; Assessed side: not informed. Ryan et al. (2017) (2): criteria used for sarcopenia diagnosis: EWGSOP; not informed. Ryan et al. (2017) (3): criteria used for sarcopenia diagnosis: IWG; Assessed side: not informed. Ryan et al. (2017) (4): criteria used for sarcopenia diagnosis: Appendicular lean mass/BMI (body mass index); Assessed side: not informed.

**FIGURE 3:**
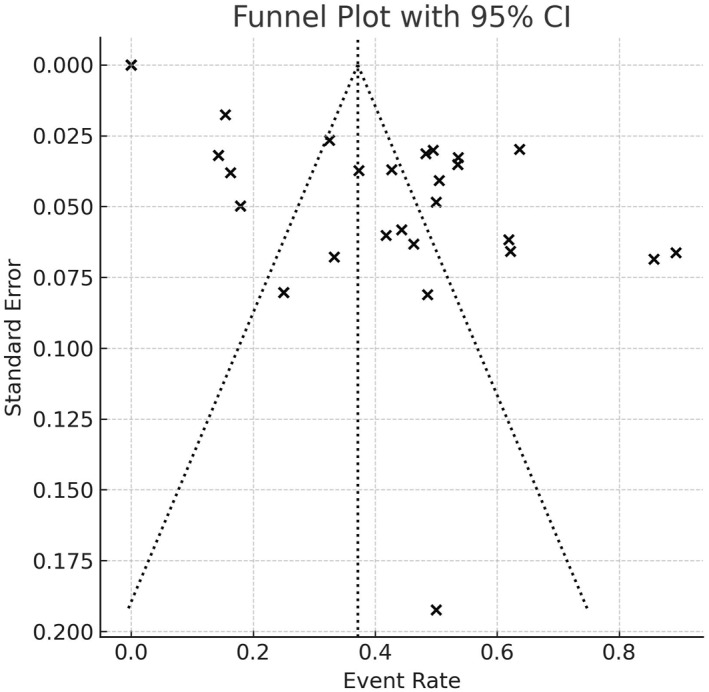
Funnel plot illustrating the relationship between event rate and study precision (standard error).

The prevalence of sarcopenia in the included studies ranged from 4.5% to 89%. Figure 2 indicates that the studies included in this review provide moderate‐quality evidence for a 37.2% prevalence of stroke‐related sarcopenia (95% CI 30.7%–44.2%; *I* ^2^ = 97%). The evidence was downgraded from high‐to moderate‐quality because of inconsistency among studies (*I* ^2^ = 97%). Figure 3 presents the funnel plot analysis of the publication bias, which can be visually observed by the signs of asymmetry in the plot. The prevalence of post‐stroke sarcopenia is based on several assessments, which differ according to their consensus. After analyzing the data, it was possible to see that most of the authors of the articles assess sarcopenia using the Asian Working Group for Sarcopenia consensus (AWGS) (Abe et al. 2023; Abe et al. 2024; Inoue et al. 2021; Jang et al. 2020; Liu et al. 2020; Maeda and Akagi 2017; Matsushita et al. 2020; Mohammed and Li 2022; Park et al. 2019; Sato et al. 2022; Shiraishi et al. 2018; Wong et al. 2022; Yao et al. 2022; Yoshimura, Bise, et al. 2018; Yoshimura, Wakabayashi, et al. 2018; Yoshimura et al. 2022a, 2022b; Nozoe et al. 2024; Ogino et al. 2024). Other studies were carried out by other consensuses, such as: the European consensus (EWGSOP), six articles (Chang et al. 2020; Landi et al. 2012; Shimizu et al. 2022; Vahlberg et al. 2016; Yu et al. 2014); three articles used more than one consensus to determine the presence of sarcopenia (Li et al. 2022; Matsushita et al. 2019; Ryan et al. 2017); two articles used the consensus Janssen and ESPEN and EASO (Yoshimura et al. 2022a, 2022b; Imamura et al. 2023).

**FIGURE 4:**
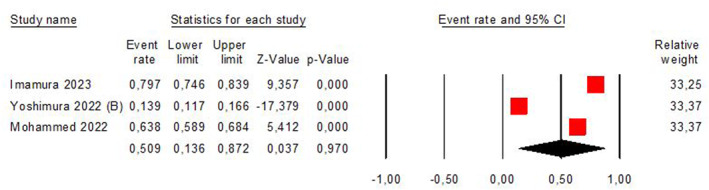
The pooled prevalence of stroke‐related sarcopenia, considering the screening.

Figure 4 shows that included studies in the present review provide low‐quality evidence that the screening of stroke‐related sarcopenia was 42.4% (95% CI 13.0%–78.5%; *I* ^2^ = 99%). The evidence was downgraded from high‐to moderate‐quality because inconsistency among studies. One article used BMI for screening (Yoshimura et al. 2022a, 2022b) and three articles carried out SARC‐F screening (Mohammed and Li 2022; Imamura et al. 2023; Galvão et al. 2024).

Table 3 reports the subgroup analysis of the prevalence of sarcopenia. It was observed a higher prevalence of sarcopenia on chronic phase after stroke, when compared to acute phase (pooled prevalence = 38.5 95% CI 23.4%–56.3%; *I* ^2^ = 94.4% vs. pooled prevalence = 38.1 95% CI 27.0%–50.6%; *I* ^2^ = 97.9%, respectively). Regarding the side of assessment, it was observed that a higher prevalence of sarcopenia in the paretic side, when compared to non‐paretic side (pooled prevalence = 87.4 95% CI 75.8%–93.9%; *I* ^2^ = 0% vs. pooled prevalence = 37.0 95% CI 29.2%–45.6%; *I* ^2^ = 69.5%, respectively). AWGS was able to detect more cases of sarcopenia (44.9%), when compared to EWGSOP (32.2%) (95% CI 37.0%–53.1%; *I* ^2^ = 95.1% vs. 95% CI 18.4%–49.9%; *I* ^2^ = 95.1%, respectively).

**TABLE 3:** Subgroup analysis of the prevalence of sarcopenia.

Subgroup	Number of studies	Events	Total	Pooled prevalence	95% CI	*I* ^2^ (%)	*p*
Stage after stroke							
Acute	10	1455	3732	38.1	27.0–50.6	97.9	< 0.01
Chronic	6	217	978	38.5	23.4–56.3	94.4	< 0.01
Side of assessment							
Paretic	1	24	28	87.4	75.8–93.9	0	< 0.01
Non‐paretic	21	1815	4915	37.0	29.2–45.6	69.5	< 0.01
Criteria used for the diagnosis							
AWGS	19	2083	4660	44.9	37.0–53.1	95.1	< 0.01
EWGSOP	8	389	1059	32.2	18.4–49.9	96.3	< 0.01

Abbreviations: AWGS = Asian Working Group for Sarcopenia; EWGSOP = European Working Group for Sarcopenia in Older People.

## 5 Discussion

The present systematic review found of 37.2% (diagnosis) and 42.4% (screening) prevalence of stroke related sarcopenia (moderate‐quality evidence), pooling 29 (*n* = 7013) and 4 (*n* = 1499) studies, respectively, showing a slight difference when compared to the 42% diagnosis prevalence found in the previous study of Su et al. (2020). However, it is important to notice that the previous review included only seven studies (*n* = 1695 stroke individuals), which might explain the differences between the systematic reviews. This difference between the number of studies included can be due the inclusion of the last 3 years publications and the additional databases and gray literature searches. This high prevalence is to be expected and is proportional to the degree of musculoskeletal deficit in post‐stroke individuals as sarcopenia becomes part of the disease related muscle atrophy (Li et al. 2020). Also, prestroke sarcopenia and low muscle mass are associated with a worse functional prognosis after the onset of the disease (Abe et al. 2023).

This review shows that the most widely used diagnostic criteria for sarcopenia is the AWGS consensus (58% of the included studies). Subgroups analysis of the prevalence of sarcopenia demonstrated that AWGS was able to detect the presence of sarcopenia more than EWGSOP (44.9% vs. 32.2%). Future studies that compared the accuracy of both consensus in individuals after stroke are necessary, in order to stablish the best instrument to this population.

It is known that there is a lack of consensus on defining a worldwide used definition and diagnostic criteria for sarcopenia, but it is also important to consider that different populational characteristics require individualized analysis of each population. There is a huge discrepancy in the number of publications on sarcopenia when we compare the seven continents. The latest review included seven Asian studies, and only one American study (Su et al. 2020). On the other hand, the present study found that 80.6% of the studies were carried out on the Asian continent, 9.6% in America, 6.4% in Europe, and one study included individuals from Asia and Africa. Given that disparity, further studies in Oceania, Africa, America and Europe are needed for a better global overview, since it is well established that the burden of stroke is different depending on the country (GBD 2019 Stroke Collaborators et al. 2021).

In the present review, the included studies used a variety of tools to measure body mass, in the same way as the latest systematic review, which may interfere with the results (Su et al. 2020). On both studies the body mass tools used were appendicular lean mass/height^2^ (ALM/ht^2^), and SMI by bioelectrical impedance analysis (BIA) and dual energy x‐ray absorptiometry (DXA) (Su et al. 2020). The assessment of main handgrip strength was utilized, although seven studies did not assess this variable. In the previous systematic review, most of the studies investigated the handgrip strength (5 out of 7) (Su et al. 2020). In this sense, it was previously found that handgrip strength is associated with risks of new‐onset stroke (Li et al. 2023), limitations in performing bimanual activities (Basílio et al. 2016) and reflects the global upper limb strength of individuals after stroke (Martins et al. 2015; Ekstrand et al. 2016). Yan et al. (2025) noted that high heterogeneity in screening and diagnostic tools compromises the analysis of pooled estimates, since it may lead to both overestimation and underestimation of the prevalence of sarcopenia.

This systematic review included five studies that have used a screening for sarcopenia meanwhile the last meta‐analysis only considered diagnosed sarcopenia (Su et al. 2020). The instruments used for screening present high sensitivity and must be widely available, it is easy to use, efficient, non‐invasive and inexpensive (Ackermans et al. 2022). One of the studies included that assessed probable sarcopenia used the SARC‐F criteria, which includes five components: Strength (S), Assistance walking (A), Rising from a chair (R), Climbing stairs (C), and Falls (F) (Imamura et al. 2023).

Although the literature reports that hospitalized individuals are more likely to lose muscle mass (Martins et al. 2015), it was observed a similar prevalence of sarcopenia on acute (38.1%) and chronic (38.5%) phase after stroke. Most of the individuals included in this systematic review were in an in‐hospital setting (85.7%), in accordance with Su et al. (2020), that found 97.7% of the individuals included were in‐hospital setting. Therefore, more studies on the prevalence of sarcopenia in post stroke individuals in the community in general are needed, since muscle mass loss is also observed in the chronic phase.

It is important to notice that in the included studies, the assessments were performed on the non‐paretic limb or on both sides, being the higher value mostly considered. However, neglecting to assess the paretic side may lead to an underdiagnosis of the condition, once the paretic limb can already present intramuscular changes resulting from the stroke. It is well reported in the literature that post‐stroke individuals show an important reduction of the paretic thigh muscle area and volume when compared to the non‐paretic limb, also having an increased intramuscular fat and decreased strength (Ryan et al. 2011; Cauraugh and Kim 2003). This highlights that assessing the paretic side can screen sarcopenia in a more efficient and quick way, once it considers the neuromuscular factors of stroke related sarcopenia.

Hemiparesis is a classic and frequent symptom after stroke and should be taken into consideration when screening for and diagnosing sarcopenia in this population, as with reduced strength, the paretic limb is used less or even neglected (Carda et al. 2013). Therefore, the loss of lean mass due to disuse should be considered, as it can influence the accuracy of the diagnosis of sarcopenia by not representing the whole body (Cauraugh and Kim 2003). The pooled prevalence demonstrated that, when considered the non‐paretic side, the prevalence was less than half of the paretic side (37% and 87.4%, respectively). Thus, these findings highlight the importance of the sarcopenia assessment considering the paretic side, since the early diagnosis allows specific treatment to be implemented.

High heterogeneity was observed in all metanalyses, except for the subgroup analysis of the sarcopenia diagnosis considering the paretic side (0%). The high heterogeneity could be explained by clinical heterogeneity, variability in study designs which were included, variability in the participants (phase after stroke, setting), and the lack of standardization of instruments used for screening and diagnoses. Thus, future studies should be developed to investigate all aspects related to sarcopenia.

This study has a notable strength considering the presented results and analysis. Prevalence data may help clinicians understand the occurrence of sarcopenia in individuals after stroke, which facilitates the planning of resource allocation and support prevention strategies. The standardization of screening and diagnostic instruments is central to fully understanding the burden of the disease, guiding future public health policies. However, there are several limitations to this review. First, we did not eliminate articles with poor quality owing to the limited number of studies available. Secondly, the metanalysis of the included studies presented high heterogeneity, which can interfere with the obtained results. The lack of consensus regarding the assessment methods and tools can also lead to some biased results. Furthermore, this analysis did not stratify prevalence by age, sex or stroke subtypes, and most included studies did not describe disease duration or stroke severity of participants. Thus, future work might further enhance prevalence estimates by incorporating these variables on data stratification. At last, almost 81% of the included studies were conducted in Asia, which may also involve a geographical bias. Thus, we recommend some caution on the interpretation of the results of this review.

## 6 Conclusion

It was observed that there was a high prevalence of probable and diagnosed sarcopenia in post‐stroke individuals, although there was a large variety of instruments used for assessing this condition. The lack of unified consensus for assessing sarcopenia in post‐stroke individuals is an important factor that needs attention. Although sarcopenia is present in all phases of stroke, most studies were developed during acute and subacute phases. This emphasized the need for studies to investigate all aspects related to sarcopenia.

## 7 Implications for Physiotherapy Practice

– More than 70% of individuals with stroke related sarcopenia were in hospital settings and wards.
– A higher prevalence of sarcopenia was observed on the paretic side when compared to the non‐paretic side.
– A higher prevalence of sarcopenia was observed in the chronic phase after stroke, when compared to the acute phase.

## Funding

This work was supported by the Conselho Nacional de Desenvolvimento Científico e Tecnológico (CNPq, Grant CNPq/MCTI/FNDCT 22/2024), Coordenação de Aperfeiçoamento de Pessoal de Nível Superior (CAPES), Pró‐reitoria de Pesquisa da Universidade Federal de Minas Gerais (PRPq/UFMG), and Fundação de Amparo à Pesquisa do Estado de Minas Gerais (FAPEMIG).

## Ethics Statement

The authors have nothing to report.

## Consent

The authors have nothing to report.

## Conflicts of Interest

The authors declare no conflicts of interest.

## Data Availability

The authors have nothing to report.

## References

[pri70134-bib-0001] Abe, T. , Y. Yoshimura , Y. Sato , F. Nagano , and A. Matsumoto . 2023. “Validity of Sarcopenia Diagnosis Defined by Calf Circumference for Muscle Mass to Predict Functional Outcome in Patients With Acute Stroke.” Archives of Gerontology and Geriatrics 105: 104854. 10.1016/j.archger.2022.104854. 36370655

[pri70134-bib-0002] Abe, T. , Y. Yoshimura , Y. Sato , F. Nagano , and A. Matsumoto . 2024. “Sarcopenia as a Robust Predictor of Readmission Within Six Months Among Individuals Experiencing Acute Stroke.” Annals of Geriatric Medicine and Research 28, no. 3: 307–314. 10.4235/agmr.24.0017. 38600867 PMC11467510

[pri70134-bib-0003] Ackermans, L. L. G. C. , J. Rabou , M. Basrai , et al. 2022. “Screening, Diagnosis and Monitoring of Sarcopenia: When to Use Which Tool?” Clinical Nutrition ESPEN 48: 36–44. 10.1016/j.clnesp.2022.01.027. 35331514

[pri70134-bib-0004] Bahat, G. , T. Erdoğan , and B. İlhan . 2022. “SARC‐F and Other Screening Tests for Sarcopenia.” Current Opinion in Clinical Nutrition and Metabolic Care 25, no. 1: 37–42. 10.1097/MCO.0000000000000801. 34861669

[pri70134-bib-0005] Basílio, M. L. , I. de Faria‐Fortini , J. C. Polese , A. A. Scianni , C. D. Faria , and L. F. Teixeira‐Salmela . 2016. “Handgrip Strength Deficits Best Explain Limitations in Performing Bimanual Activities After Stroke.” Journal of Physical Therapy Science 28, no. 4: 1161–1165. 10.1589/jpts.28.1161. 27190447 PMC4868207

[pri70134-bib-0006] Bellelli, G. , A. Zambon , S. Volpato , et al. 2018. “The Association Between Delirium and Sarcopenia in Older Adult Patients Admitted to Acute Geriatrics Units: Results From the GLISTEN Multicenter Observational Study.” Clinical Nutrition 37, no. 5: 1498–1504. 10.1016/j.clnu.2017.08.027. 28918171

[pri70134-bib-0007] Botö, S. , D. J. Buvarp , P. O. Hansson , K. S. Sunnerhagen , and C. U. Persson . 2021. “Physical Inactivity After Stroke: Incidence and Early Predictors Based on 190 Individuals in a 1‐Year Follow‐Up of the Fall Study of Gothenburg.” Journal of Rehabilitation Medicine 53, no. 9 (September): jrm00224. 10.2340/16501977-2852. 34121128 PMC8638731

[pri70134-bib-0008] Carda, S. , C. Cisari , and M. Invernizzi . 2013. “Sarcopenia or Muscle Modifications in Neurologic Diseases: A Lexical or Patophysiological Difference?” European Journal of Physical and Rehabilitation Medicine 49, no. 1: 119–130. https://pubmed.ncbi.nlm.nih.gov/23575206/. 23575206

[pri70134-bib-0009] Cauraugh, J. H. , and S. B. Kim . 2003. “Chronic Stroke Motor Recovery: Duration of Active Neuromuscular Stimulation.” Journal of Neurological Sciences 215, no. 1‒2: 13–19. 10.1016/s0022-510x(03)00169-2. 14568122

[pri70134-bib-0010] Chang, K. V. , W. T. Wu , K. C. Huang , and D. S. Han . 2020. “Segmental Body Composition Transitions in Stroke Patients: Trunks are Different From Extremities and Strokes are as Important as Hemiparesis.” Clinical Nutrition 39, no. 6: 1968–1973. 10.1016/j.clnu.2019.08.024. 31522786

[pri70134-bib-0011] Cruz‐Jentoft, A. J. , G. Bahat , J. Bauer , et al. 2019. “Sarcopenia: Revised European Consensus on Definition and Diagnosis.” Age and Ageing 48, no. 1: 16–31. 10.1093/ageing/afy169. 30312372 PMC6322506

[pri70134-bib-0012] Diz, J. B. , A. A. Leopoldino , B. S. Moreira , et al. 2017. “Prevalence of Sarcopenia in Older Brazilians: A Systematic Review and Meta‐Analysis.” Geriatrics and Gerontology International 17, no. 1: 5–16. 10.1111/ggi.12720. 26799062

[pri70134-bib-0013] Dos Reis, A. H. S. , A. L. M. de Oliveira , C. Fritsch , J. Zouch , P. Ferreira , and J. C. Polese . 2023. “Usefulness of Machine Learning Softwares to Screen Titles of Systematic Reviews: A Methodological Study.” Systematic Reviews 12, no. 1: 68. 10.1186/s13643-023-02231-3. 37061711 PMC10105467

[pri70134-bib-0014] Ekstrand, E. , J. Lexell , and C. Brogårdh . 2016. “Grip Strength is a Representative Measure of Muscle Weakness in the Upper Extremity After Stroke.” Topics in Stroke Rehabilitation 23, no. 6: 400–405. 10.1080/10749357.2016.1168591. 27145212

[pri70134-bib-0015] Evans, W. J. 2010. “Skeletal Muscle Loss: Cachexia, Sarcopenia, and Inactivity.” American Journal of Clinical Nutrition 91, no. 4: 1123S–1127S. 10.3945/ajcn.2010.28608A. 20164314

[pri70134-bib-0016] Galvão, A. C. J. , C. Dias , A. L. Miranda , et al. 2024. “Stroke Related Sarcopenia in Individuals With Different Physical Activity Levels: A Cross‐Sectional Study.” Physiotherapy Research International: The Journal for Researchers and Clinicians in Physical Therapy 29, no. 2: e2084. 10.1002/pri.2084. 38529736

[pri70134-bib-0017] Gao, L. , Jiang, J. , Yang, M. , Hao, Q. , Luo, L. , and Dong, B. 2015. “Prevalence of Sarcopenia and Associated Factors in Chinese Community‐Dwelling Elderly: Comparison Between Rural and Urban Areas.” Journal of the American Medical Directors Association 16, no. 11: 1003.e1. 10.1016/j.jamda.2015.07.020. 26385304

[pri70134-bib-0018] GBD 2019 Stroke Collaborators , Stark, B. A. , C. O. Johnson , et al. 2021. “Global, Regional, and National Burden of Stroke and its Risk Factors, 1990–2019: A Systematic Analysis for the Global Burden of Disease Study 2019.” Lancet Neurology 20, no. 10: 795–820. 10.1016/S1474-4422(21)00252-0. 34487721 PMC8443449

[pri70134-bib-0019] Higgins, J. P. , and S. Green , eds. 2023. Cochrane Handbook for Systematic Reviews of Interventions, Version 6.4. Cochrane Collaboration.

[pri70134-bib-0020] Higgins, J. P. , and S. G. Thompson . 2002. “Quantifying Heterogeneity in a Meta‐Analysis.” Statistics in Medicine 21, no. 11: 1539–1558. 10.1002/sim.1186. 12111919

[pri70134-bib-0021] Imamura, M. , M. Nozoe , H. Kubo , and S. Shimada . 2023. “Association Between Premorbid Sarcopenia and Neurological Deterioration in Patients With Acute Ischemic Stroke.” Clinical Neurology and Neurosurgery 224: 107527. 10.1016/j.clineuro.2022.107527. 36455301

[pri70134-bib-0022] Inoue, T. , K. Maeda , A. Shimizu , et al. 2021. “Calf Circumference Value for Sarcopenia Screening Among Older Adults With Stroke.” Archives of Gerontology and Geriatrics 93: 104290. 10.1016/j.archger.2020.104290. 33171328

[pri70134-bib-0023] Jang, Y. , S. Im , Y. Han , H. Koo , D. Sohn , and G. Y. Park . 2020. “Can Initial Sarcopenia Affect Poststroke Rehabilitation Outcome?” Journal of Clinical Neuroscience 71: 113–118. 10.1016/j.jocn.2019.08.109. 31495657

[pri70134-bib-0024] Kortebein, P. , A. Ferrando , J. Lombeida , R. Wolfe , and W. J. Evans . 2007. “Effect of 10 Days of Bed Rest on Skeletal Muscle in Healthy Older Adults.” JAMA 297, no. 16: 1772–1774. 10.1001/jama.297.16.1772-b. 17456818

[pri70134-bib-0025] Landi, F. , R. Liperoti , A. Russo , et al. 2012. “Sarcopenia as a Risk Factor for Falls in Elderly Individuals: Results From the Ilsirente Study.” Clinical Nutrition 31, no. 5: 652–658. 10.1016/j.clnu.2012.02.007. 22414775

[pri70134-bib-0026] Lera, L. , C. Albala , H. Sánchez , et al. 2017. “Prevalence of Sarcopenia in Community‐Dwelling Chilean Elders According to an Adapted Version of the European Working Group on Sarcopenia in Older People (EWGSOP) Criteria.” Journal of Frailty and Aging 6, no. 1: 12–17. 10.14283/jfa.2016.117. 28244552

[pri70134-bib-0027] Li, G. , Y. Lu , L. Shao , et al. 2023. “Handgrip Strength is Associated With Risks of New‐Onset Stroke and Heart Disease: Results From 3 Prospective Cohorts.” BMC Geriatrics 23, no. 1: 268. 10.1186/s12877-023-03953-8. 37142986 PMC10161641

[pri70134-bib-0028] Li, S. , J. Gonzalez‐Buonomo , J. Ghuman , et al. 2022. “Aging After Stroke: How to Define Post‐Stroke Sarcopenia and are Its Risk Factors?” European Journal of Physical and Rehabilitation Medicine 58, no. 5: 683–692. 10.23736/S1973-9087.22.07514-1. 36062331 PMC10022455

[pri70134-bib-0029] Li, W. , T. Yue , and Y. Liu . 2020. “New Understanding of the Pathogenesis and Treatment of Stroke‐Related Sarcopenia.” Biomedicine & Pharmacotherapy 131: 110721. 10.1016/j.biopha.2020.110721. 32920517

[pri70134-bib-0030] Liu, G. , C. W. Zheng , and D. H. Zhou . 2020. “Study on the Incidence of Stroke‐Related Sarcopenia in Hospitalized Male Patients.” Open Access Library Journal 7, no. 12: 1–4. 10.4236/oalib.1107009.

[pri70134-bib-0031] Maeda, K. , and J. Akagi . 2017. “Cognitive Impairment is Independently Associated With Definitive and Possible Sarcopenia in Hospitalized Older Adults: The Prevalence and Impact of Comorbidities.” Geriatrics and Gerontology International 17, no. 7: 1048–1056. 10.1111/ggi.12825. 27273820

[pri70134-bib-0032] Martins, J. C. , L. T. Aguiar , E. M. Lara , L. F. Teixeira‐Salmela , and C. D. Faria . 2015. “Assessment of Grip Strength With the Modified Sphygmomanometer Test: Association Between Upper Limb Global Strength and Motor Function.” Brazilian Journal of Physical Therapy 19, no. 6: 498–506. 10.1590/bjpt-rbf.2014.0118. 26647752 PMC4668344

[pri70134-bib-0033] Matsushita, T. , S. Nishioka , S. Taguchi , and A. Yamanouchi . 2019. “Sarcopenia as a Predictor of Activities of Daily Living Capability in Stroke Patients Undergoing Rehabilitation.” Geriatrics and Gerontology International 19, no. 11: 1124–1128. 10.1111/ggi.13780. 31591820

[pri70134-bib-0034] Matsushita, T. , S. Nishioka , S. Taguchi , A. Yamanouchi , R. Nakashima , and H. Wakabayashi . 2020. “Sarcopenic Obesity and Activities of Daily Living in Stroke Rehabilitation Patients: A Cross‐Sectional Study.” Healthcare 8, no. 3: 255. 10.3390/healthcare8030255. 32781673 PMC7551564

[pri70134-bib-0035] Mohammed, M. , and J. Li . 2022. “Stroke‐Related Sarcopenia Among Two Different Developing Countries With Diverse Ethnic Backgrounds (Cross‐National Study in Egypt and China).” Healthcare 10, no. 11: 2336. 10.3390/healthcare10112336. 36421660 PMC9689982

[pri70134-bib-0036] Munn, Z. , S. Moola , K. Lisy , and D. Riitano . 2014. The Joanna Briggs Institute Reviewers' Manual 2014. The Systematic Review of Prevalence and Incidence Data. Joanna Briggs Institute.

[pri70134-bib-0037] Munn, Z. , S. Moola , K. Lisy , D. Riitano , and C. Tufanaru . 2015. “Methodological Guidance for Systematic Reviews of Observational Epidemiological Studies Reporting Prevalence and Cumulative Incidence Data.” International Journal of Evidence‐Based Healthcare 13, no. 3: 147–153. 10.1097/XEB.0000000000000054. 26317388

[pri70134-bib-0038] Nozoe, M. , H. Kubo , M. Yamamoto , et al. 2024. “Muscle Weakness is More Strongly Associated With Functional Outcomes in Patients With Stroke than Sarcopenia or Muscle Wasting: An Observational Study.” Aging Clinical and Experimental Research 36, no. 1: 4. 10.1007/s40520-023-02672-9. 38261059 PMC10806041

[pri70134-bib-0039] Ogino, T. , M. Nozoe , T. Inoue , M. Ishida , and K. Yamamoto . 2024. “Impact of Possible Sarcopenia on Functional Prognosis in Patients With Acute Stroke With Premorbid Disability.” Geriatrics and Gerontology International 24, no. 4: 359–363. 10.1111/ggi.14840. 38410028 PMC11503578

[pri70134-bib-0040] Park, J. G. , K. W. Lee , S. B. Kim , J. H. Lee , and Y. H. Kim . 2019. “Effect of Decreased Skeletal Muscle Index and Hand Grip Strength on Functional Recovery in Subacute Ambulatory Stroke Patients.” Annals of Rehabilitation Medicine 43, no. 5: 535–543. 10.5535/arm.2019.43.5.535. 31693843 PMC6835132

[pri70134-bib-0041] Park, S. , J. O. Ham , and B. K. Lee . 2014. “A Positive Association Between Stroke Risk and Sarcopenia in Men Aged ≥ 50 Years, but Not Women: Results From the Korean National Health and Nutrition Examination Survey 2008‒2010.” Journal of Nutrition, Health & Aging 18, no. 9: 806–812. 10.1007/s12603-014-0553-x.25389958

[pri70134-bib-0042] Patel, H. P. , H. E. Syddall , K. Jameson , et al. 2013. “Prevalence of Sarcopenia in community‐Dwelling Older People in the UK Using the European Working Group on Sarcopenia in Older People (EWGSOP) Definition: Findings From the Hertfordshire Cohort Study (HCS).” Age and Ageing 42, no. 3: 378–384. 10.1093/ageing/afs197. 23384705 PMC3633365

[pri70134-bib-0043] Ryan, A. S. , A. Buscemi , L. Forrester , C. E. Hafer‐Macko , and F. M. Ivey . 2011. “Atrophy and Intramuscular Fat in Specific Muscles of the Thigh: Associated Weakness and Hyperinsulinemia in Stroke Survivors.” Neurorehabilitation and Neural Repair 25, no. 9 (November‒December): 865–872. 10.1177/1545968311408920. 21734070 PMC3546168

[pri70134-bib-0044] Ryan, A. S. , F. M. Ivey , M. C. Serra , J. Hartstein , and C. E. Hafer‐Macko . 2017. “Sarcopenia and Physical Function in Middle‐Aged and Older Stroke Survivors.” Archives of Physical Medicine and Rehabilitation 98, no. 3: 495–499. 10.1016/j.apmr.2016.07.015. 27530769 PMC5305627

[pri70134-bib-0045] Sato, Y. , Y. Yoshimura , and T. Abe . 2022. “Phase Angle as an Indicator of Baseline Nutritional Status and Sarcopenia in Acute Stroke.” Journal of Stroke and Cerebrovascular Diseases 31, no. 1: 106220. 10.1016/j.jstrokecerebrovasdis.2021.106220. 34826661

[pri70134-bib-0046] Scherbakov, N. , S. von Haehling , S. D. Anker , U. Dirnagl , and W. Doehner . 2013. “Stroke Induced Sarcopenia: Muscle Wasting and Disability After Stroke.” International Journal of Cardiology 170, no. 2: 89–94. 10.1016/j.ijcard.2013.10.031. 24231058

[pri70134-bib-0047] Shimizu, A. , I. Fujishima , K. Maeda , et al. 2022. “Association Between Food Texture Levels Consumed and the Prevalence of Malnutrition and Sarcopenia in Older Patients After Stroke.” European Journal of Clinical Nutrition 76, no. 11: 1576–1582. 10.1038/s41430-022-01126-1. 35418607

[pri70134-bib-0048] Shiraishi, A. , Y. Yoshimura , H. Wakabayashi , and Y. Tsuji . 2018. “Prevalence of Stroke‐Related Sarcopenia and Its Association With Poor Oral Status in Post‐Acute Stroke Patients: Implications for Oral Sarcopenia.” Clinical Nutrition 37, no. 1: 204–207. 10.1016/j.clnu.2016.12.002. 28017450

[pri70134-bib-0049] Stroup, D. F. , J. A. Berlin , S. C. Morton , et al. 2000. “Meta‐Analysis of Observational Studies in Epidemiology: A Proposal for Reporting. Meta‐Analysis of Observational Studies in Epidemiology (MOOSE) Group.” JAMA 283, no. 15: 2008–2012. 10.1001/jama.283.15.2008. 10789670

[pri70134-bib-0050] Su, Y. , M. Yuki , and M. Otsuki . 2020. “Prevalence of Stroke‐Related Sarcopenia: A Systematic Review and Meta‐Analysis.” Journal of Stroke and Cerebrovascular Diseases 29, no. 9: 105092. 10.1016/j.jstrokecerebrovasdis.2020.105092. 32807486

[pri70134-bib-0051] Terracciano, L. , J. Brozek , E. Compalati , and H. Schünemann . 2010. “GRADE System: New Paradigm.” Current Opinion in Allergy and Clinical Immunology 10, no. 4: 377–383. 10.1097/ACI.0b013e32833c148b. 20610980

[pri70134-bib-0052] Tsao, C. W. , A. W. Aday , Z. I. Almarzooq , et al. 2023. “Heart Disease and Stroke Statistics‐2023 Update: A Report From the American Heart Association.” Circulation 147, no. 8: e93–e621. 10.1161/CIR.0000000000001123. 36695182 PMC12135016

[pri70134-bib-0053] Vahlberg, B. , L. Zetterberg , B. Lindmark , K. Hellström , and T. Cederholm . 2016. “Functional Performance, Nutritional Status, and Body Composition in Ambulant Community‐Dwelling Individuals 1‒3 Years After Suffering From a Cerebral Infarction or Intracerebral Bleeding.” BMC Geriatrics 16, no. 1: 48. 10.1186/s12877-016-0226-1. 26895855 PMC4759921

[pri70134-bib-0054] Wong, H. J. , S. Harith , P. L. Lua , and K. A. Ibrahim . 2022. “Possible Sarcopenia and Its Association With Nutritional Status, Dietary Intakes, Physical Activity and Health‐Related Quality of Life Among Older Stroke Survivors.” Annals of Geriatric Medicine and Research 26, no. 2: 162–174. 10.4235/agmr.22.0033. 35726561 PMC9271400

[pri70134-bib-0055] Yan, H. , J. Li , L. Xian , Y. Li , S. Li , and Q. Wen . 2025. “Risk Factors of Stroke‐Related Sarcopenia: A Systematic Review and Meta‐Analysis.” Frontiers in Aging Series 6: 1452708. 10.3389/fragi.2025.1452708.January 31 PMC11833335 39967995

[pri70134-bib-0056] Yang, M. , X. Hu , L. Xie , et al. 2018. “Screening Sarcopenia in Community‐Dwelling Older Adults: SARC‐F vs SARC‐F Combined With Calf Circumference (SARC‐CalF).” Journal of the American Medical Directors Association 19, no. 3: 277.e1–277.e8. 10.1016/j.jamda.2017.12.016. 29477774

[pri70134-bib-0057] Yao, R. , L. Yao , A. Rao , et al. 2022. “Prevalence and Risk Factors of Stroke‐Related Sarcopenia at the Subacute Stage: A Case Control Study.” Frontiers in Neurology 13: 899658. 10.3389/fneur.2022.899658. 36003303 PMC9393529

[pri70134-bib-0058] Yoshimura, Y. , T. Bise , F. Nagano , et al. 2018. “Systemic Inflammation in the Recovery Stage of Stroke: Its Association With Sarcopenia and Poor Functional Rehabilitation Outcomes.” Progress in Rehabilitation Medicine 18, no. 3: 20180011. 10.2490/prm.20180011. PMC7365223 32789236

[pri70134-bib-0059] Yoshimura, Y. , H. Wakabayashi , T. Bise , and M. Tanoue . 2018. “Prevalence of Sarcopenia and Its Association With Activities of Daily Living and Dysphagia in Convalescent Rehabilitation Ward Inpatients.” Clinical Nutrition 37, no. 6 pt. A: 2022–2028. 10.1016/j.clnu.2017.09.009. 28987469

[pri70134-bib-0060] Yoshimura, Y. , H. Wakabayashi , F. Nagano , et al. 2022a. “Chair‐Stand Exercise Improves Sarcopenia in Rehabilitation Patients After Stroke.” Nutrients 14, no. 3: 461. 10.3390/nu14030461. 35276820 PMC8840470

[pri70134-bib-0061] Yoshimura, Y. , H. Wakabayashi , F. Nagano , et al. 2022b. “The Applicability of the ESPEN and EASO‐Defined Diagnostic Criteria for Sarcopenic Obesity in Japanese Patients After Stroke: Prevalence and Association With Outcomes.” Nutrients 14, no. 19: 4205. 10.3390/nu14194205. 36235857 PMC9570818

[pri70134-bib-0062] Yu, R. , M. Wong , J. Leung , J. Lee , T. W. Auyeung , and J. Woo . 2014. “Incidence, Reversibility, Risk Factors and the Protective Effect of High Body Mass Index Against Sarcopenia in Community‐Dwelling Older Chinese Adults [Published Correction Appears in *Geriatr Gerontol Int*. 14(3):730].” Geriatrics and Gerontology International 14 suppl 1: 15–28. 10.1111/ggi.12220. 24450557

